# Single-Stranded DNA Catalyzes Hybridization of PCR-Products to Microarray Capture Probes

**DOI:** 10.1371/journal.pone.0102338

**Published:** 2014-07-15

**Authors:** Simon Dally, Steffen Rupp, Karin Lemuth, Stefan C. Hartmann, Ekkehard Hiller, Susanne M. Bailer, Cornelius Knabbe, Jan Weile

**Affiliations:** 1 Institute for Laboratory and Transfusion Medicine, Heart and Diabetes Center North Rhine-Westphalia, Bad Oeynhausen, Germany; 2 Department of Molecular Biotechnology, Fraunhofer Institute for Interfacial Engineering and Biotechnology, Stuttgart, Germany; University of North Carolina at Charlotte, United States of America

## Abstract

Since its development, microarray technology has evolved to a standard method in the biotechnological and medical field with a broad range of applications. Nevertheless, the underlying mechanism of the hybridization process of PCR-products to microarray capture probes is still not completely understood, and several observed phenomena cannot be explained with current models. We investigated the influence of several parameters on the hybridization reaction and identified ssDNA to play a major role in the process. An increase of the ssDNA content in a hybridization reaction strongly enhanced resulting signal intensities. A strong influence could also be observed when unlabeled ssDNA was added to the hybridization reaction. A reduction of the ssDNA content resulted in a massive decrease of the hybridization efficiency. According to these data, we developed a novel model for the hybridization mechanism. This model is based on the assumption that single stranded DNA is necessary as catalyst to induce the hybridization of dsDNA. The developed hybridization model is capable of giving explanations for several yet unresolved questions regarding the functionality of microarrays. Our findings not only deepen the understanding of the hybridization process, but also have immediate practical use in data interpretation and the development of new microarrays.

## Introduction

The DNA-microarray technology is a well-established method for the analysis of DNA, hence, forming the basis of a broad range of biological and biotechnological applications. All of them base on the hybridization of DNA target sequences to probes grafted onto a solid support. However, the underlying mechanism of the hybridization process is still not completely understood, and some observed effects, like weak or missing probe signals, are not satisfyingly explained. The data interpretation is restricted to empirical and statistical methods [1], [2].

In many applications, the targets in the sample DNA are amplified by a multiplex-PCR prior to hybridization. The bulk of these products consists of double-stranded DNA. Whereas the hybridization of single-stranded DNA to microarray probes was intensively investigated and characterized in recent years [3], the hybridization mechanism of double-stranded DNA to capture probes has not yet been elucidated clearly. Since the bases of a double strand are already bound to each other, they should not be accessible for further hybridization reactions with a probe. The formation of DNA-triplex structures is considered unlikely under typically used hybridization conditions [4]. The most likely mechanism is that the hybridization process begins by the formation of a transient nucleation complex that is built by interaction of very few bases, which then expands through a zippering process. However, the rate of spontaneous strand separation within the PCR-product, which would be necessary to generate a starting point, is very low under hybridization conditions typically used [5].

Theoretically, double stranded PCR-products can hybridize either with their sense-strand (formed by elongation of the forward primer) to their respective antisense probe or with their antisense-strand (formed by elongation of the reverse primer) to their respective sense probe, respectively. Thereby, hybridization to both, sense and antisense probes, should be detectable. However, most of the time only one of the two probes shows a positive signal [6], [7]. This poses an ambiguity which has not yet been understood, although, sometimes steric hindrance or the secondary structure of PCR-products is assumed to cause weak signals [7], [8]. Furthermore, we observed that the preference of a PCR-product to hybridize to the sense or to the antisense probe may switch under certain conditions, such as a modulation of the primer concentration in the precedent PCR. The cause of this switch is unclear so far.

Our aim in the present study was to analyze the hybridization mechanism of PCR-products to microarray capture probes in order to clarify the ambiguities mentioned above. For that purpose, we characterized the hybridization behavior of PCR-products in different experimental settings and tried to determine the underlying molecular mechanism.

## Material and Methods

### PCR conditions

A section of the gene *dfrA1*, accessible under no. CU459141 at *Genbank* (http://www.ncbi.nlm.nih.gov/genbank/), was used as model target. Amplification was conducted with the primers dfrA1_F (GAATGGAGTTATCGGGAATGGC) and dfrA1_R (CCCACCACCTGAAACAATGAC). Each PCR contained 0.02 U/µl Phusion Polymerase (Thermo Scientific), 1× Phusion GC reaction Buffer, 30 µM of each desoxynucleotide triphosphate (dNTP), 1 ng/µl template DNA, and a primer concentration dependent on the experimental setting, respectively. PCR-products intended for hybridization experiments were labeled by replacing 40% of the dCTP by Cy3-coupled dCTP. The reaction was started with an initial denaturation step at 97°C for 2 min. The cycle reactions consisted of 35 cycles of 30 s at 97°C, 30 s at 60°C, and 45 s at 72°C, followed by a final extension at 72°C for 5 min.

### Template DNA

The strain *Acinetobacter baumannii* AYE obtained by the American Type Culture Collection (ATCC-BAA-1717) was cultured in Luria–Bertani broth at 37°C for approximately 20 h. Its DNA was isolated using the DNeasy Blood & Tissue Kit (Qiagen) according to the manufacturer's instructions for gram-negative bacterial cultures. Quality and quantity of the isolated DNA were analyzed spectrophotometrically using a Nanodrop spectrophotometer (Thermo Scientific).

### Synthetic single-stranded DNA

Pure sense strand of the target section of the *dfrA1* gene was ordered at Metabion International AG. The sequence of the 268 bases long ssDNA derives from the Genbank entry HM036078 from position 18 to 285. The DNA was purified and quality checked by high-pressure liquid chromatography (HPLC).

### Capture probes

Seven capture probes for the detection of the *dfrA1* fragment were designed using the Geneious Pro 5.3.6 software. The corresponding melting temperature T_m_ of each probe was calculated by OligoAnalyzer 3.1 (Integrated DNA Technologies). All probes were ordered from Metabion International AG in sense and antisense orientation. Each Probe was synthesized with a (T)_14_ spacer at the 5′ end with an amino-modification at the C6 atom of the 5′ terminal T. Uniqueness of each probe sequence within the Genbank database was confirmed by BLAST analysis (http://www.ncbi.nlm.nih.gov/BLAST/) using default settings.

### Probe position score

The relative position of a probe's binding site within its respective target PCR-product can be displayed by the probe position score (PPS). It is calculated by dividing the distance of the probe binding site to the forward primer binding site through the length of the PCR-product less the length of both primers and the probe and multiplied with 100. According to this formula, the score has a value of 0 if the probe is adjacent to the forward primer and a value of 100 if it is adjacent to the reverse primer, respectively. Characteristics of probes used in this study, including PPS, are displayed in Table 1.

**Table 1:** Properties of microarray capture probes.

Name	T_m_ [°C]	Position [bp]	PPS	Sequence
Probe 1	57.5	22–43	1	CCCTGATATTCCATGGAGTGCC
Probe 2	58.1	40–59	9	TGCCAAAGGTGAACAGCTCC
Probe 3	57.2	91–113	33	GTTGGTTGGACGCAAGACTTTTG
Probe 4	57.4	116–137	45	TCAATGGGAGCATTACCCAACC
Probe 5	58.1	142–165	58	GTATGCGGTCGTAACACGTTCAAG
Probe 6	55.5	162–190	68	CAAGTTTTACATCTGACAATGAGAACGTA
Probe 7	55.2	200–227	86	CCATCAATTAAAGATGCTTTAACCAACC
neg. control	57.2	-	-	ACCCATCCGTTACGGCAAAA

Every probe is listed in sense orientation with its respective calculated melting temperature, relative position within the 268 bp section of the *dfrA1* gen, resulting probe position score (PPS), and sequence (without (T)_14_ spacer).

### Microarray production

Capture probes were diluted in spotting buffer (1∶1 mixture of Nexterion Spot I and Nexterion Spot modified; Schott Nexterion) to a concentration of 50 µM and spotted in duplicates on an epoxy-functionalized Nexterion Slide E (Schott Nexterion) using a Microgrid II Spotter 610 arraying system (Bio Robotics) with SMP3 pins (Arrayit Corporation). The diameter of each spot was about 80 µm and the distance between two spots about 140 µm. The covalent coupling of the probes to the functionalized glass surface took place in a 60 min incubation step at 70°C directly after the printing process. Blocking of the slides was conducted immediately before use according to the manufacturer's instruction.

### Microarray hybridization

Each hybridization experiment was conducted at least twice to reduce statistical noise. The solution loaded on the array to start the hybridization contained 30 µl PCR-product and 15 µl hybridization buffer resulting in a final concentration of 600 mM NaCl, 60 mM trisodium citrate, and 0.1% sodium dodecyl sulfate. 45 µl hybridization mixture were transferred on the array using a LifterSlip (Earie Scientific Company) and incubated for 1 h at 58°C. After hybridization, the slides were washed in 3 steps: 10 min in 0.3 M NaCl, 30 mM sodium citrate, 0.2% SDS, then 10 min in 0.3 M NaCl, 30 mM sodium citrate, and then 10 min in 30 mM NaCl, 3 mM sodium citrate, respectively. The probe signals of the arrays were detected with the fluorescence scanner Axon GenePix 4300A (MDS Analytical Technologies GmbH) using a 532 nm laser for the excitation of the Cy3 fluorophor and the integrated filter (550 nm–600 nm) for the purification of emitted light. The output of the scanner was a 16-bit TIFF file, which could be analyzed with the integrated GenePix Pro software. The software automatically identified probe spots and calculated the local background corrected signal.

## Results

**Figure 1:**
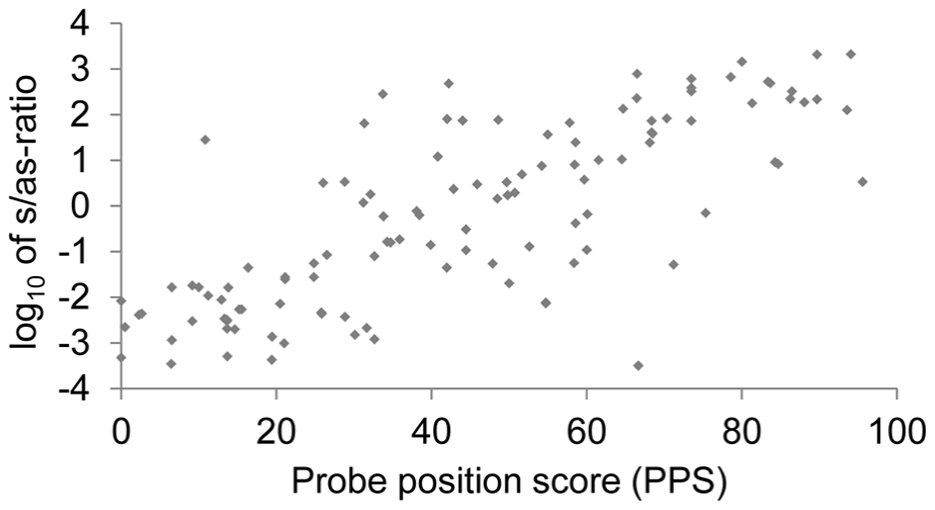
Correlation of probe signal intensity and relative probe position. Measured s/as-ratio of 115 probe pairs in logarithmic scale after hybridization of their respective specific target in relation to their probe position score.

The relative position of a probe within the sequence of its target strongly influences whether the target hybridizes to the sense or the antisense configuration of the probe [8]. In order to clarify the cause of this phenomenon, we measured the preference of 77 different PCR-products for a specific probe configuration. The products were amplified in a multiplex-PCR and hybridized altogether on a DNA-microarray, which was previously published [9]. The array contained 115 pairs of sense and antisense probes with different position scores (PPS), which were specific for these PCR-products. The ratio of the signal intensities of sense and antisense probes (s/as-ratio) for each probe pair was calculated and correlated with their probe position score. The generated data are shown in Figure 1. It could be observed that in general probe pairs in the vicinity of the forward primer binding site (PPS<50) show a low s/as-ratio and those in the vicinity of the reverse primer binding site (PPS>50) show a high s/as-ratio. The universal validity of this rule for almost all probe-target combinations indicated a sequence independent cause for the selective hybridization of PCR-products to a specific probe configuration.

**Figure 2:**
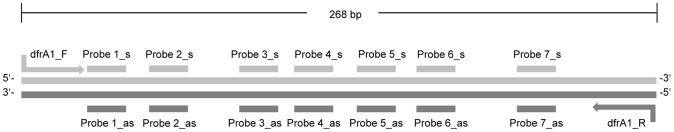
Position and nomenclature of the primers and probes within their target gene *dfrA1*. The sense strand, forward primer, and probes in sense configuration (s) are displayed in light grey, whereas antisense stand, reverse primer, and probes in antisense configuration (as) are displayed in dark grey, respectively.

**Figure 3:**
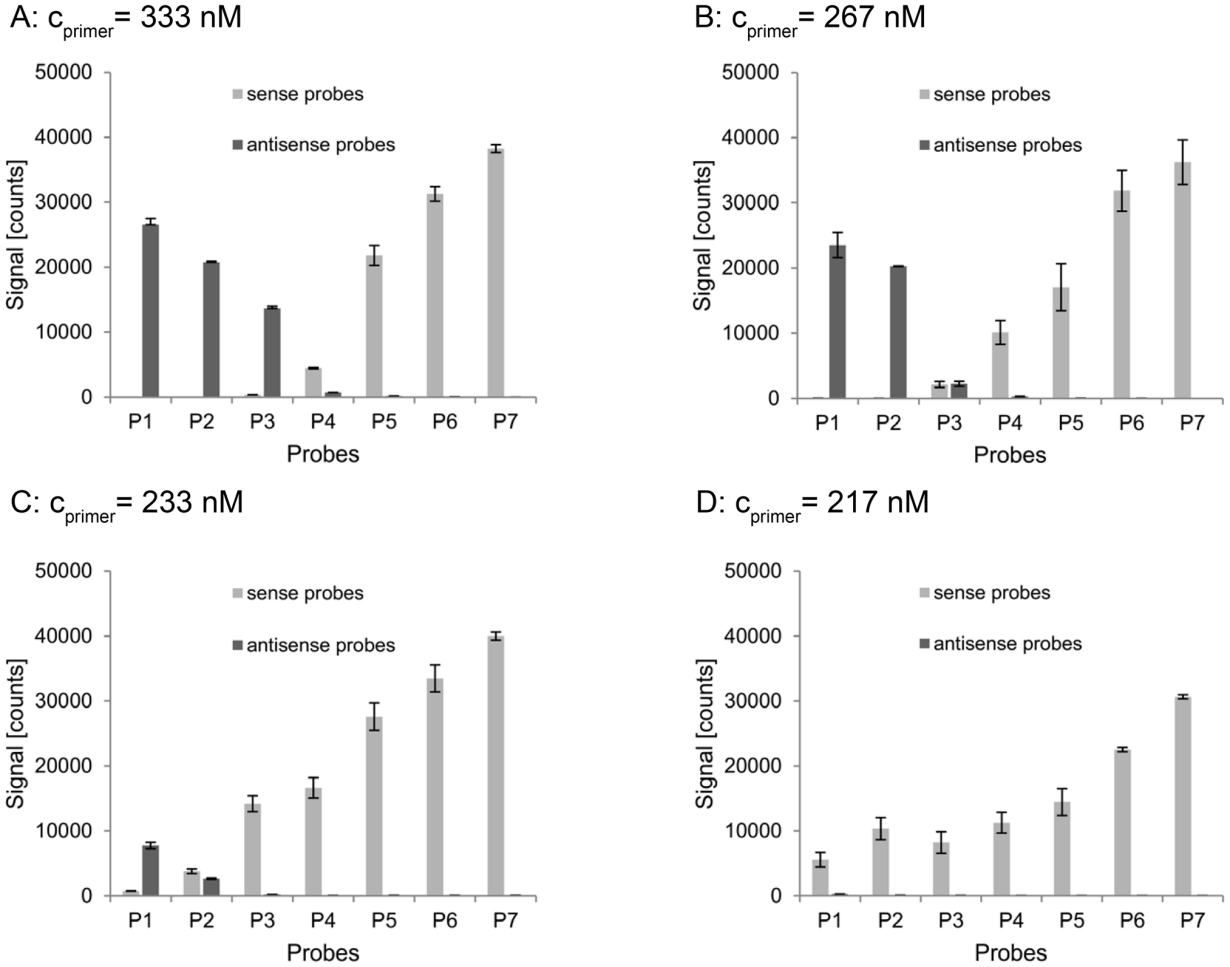
Hybridization of products of four equimolar PCRs to seven probe pairs. Signal intensities of the seven *dfrA1* specific probe pairs (P1–P7) in sense and antisense configuration after hybridization of the *dfrA1* PCR-product, which was amplified with four different primer concentrations.

**Figure 4:**
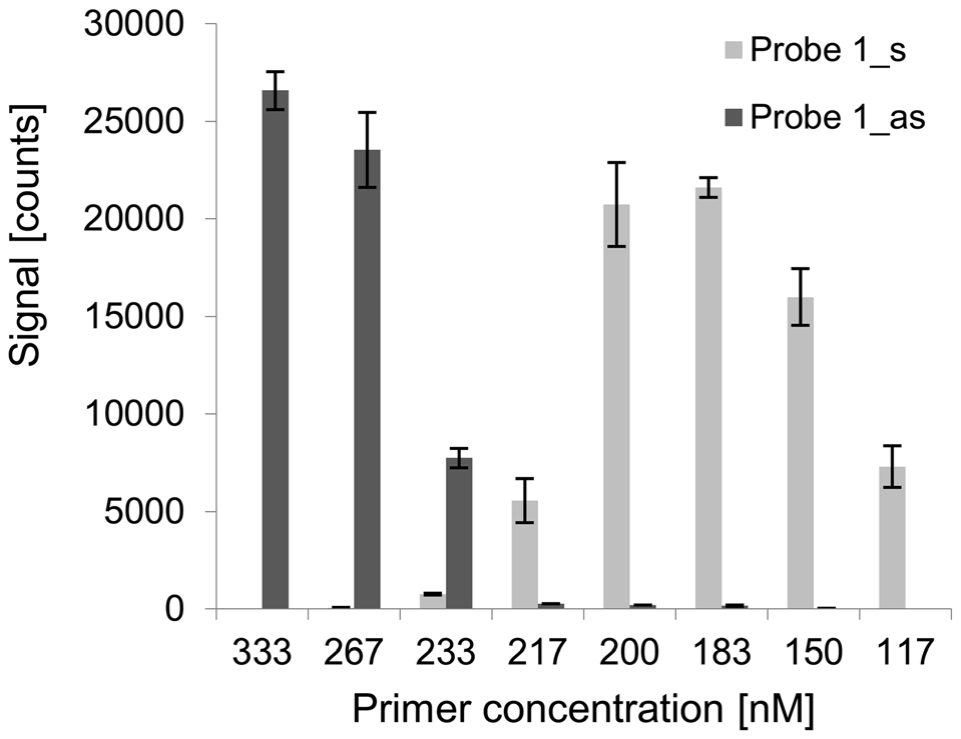
Hybridization of products of eight equimolar PCRs to probe pair 1. Signal intensities of probe1_s and probe1_as after hybridization of the *dfrA1* PCR-product, which was amplified with eight different primer concentrations.

Previous experiments had indicated that not only the PPS of a probe, but also the primer concentration in the foregoing PCR has a significant influence on the s/as-ratio of probe pairs. For detailed analysis of this effect, the hybridization behavior of the single PCR-product *dfrA1*, generated with different primer concentrations, was examined. We designed seven different probe pairs, which were specific for the gene *dfrA1*. Their binding sites were distributed over the amplified region of the *dfrA1* gene (see Figure 2). The 268 bp section of the *dfrA1* gene was amplified in four equimolar singleplex-PCRs containing 333 nM, 267 nM, 233 nM, and 217 nM of each primer. Each product was hybridized on the array, which contained the 7 specific probe pairs. The resulting signal intensities are shown in Figure 3. Again, probes with a low PPS showed a low s/as-ratio and those with a high PPS showed a high s/as-ratio, respectively. Nevertheless, comparably low primer concentrations in the PCR reaction turned out to influence the probe configuration selectivity of the PCR-product. The signal of probe pairs 1–3 shifted from the antisense to the sense probe. Four further tests with even lower primer concentrations (200 nM, 183 nM, 150 nM, and 117 nM) were conducted. Results of the eight experiments with different primer concentrations are exemplarily shown for probe pair 1 (Figure 4). The hybridization behavior of the *dfrA1* PCR-product changed dramatically in terms of its probe configuration preference when the primer concentration in the PCR was modified. A decrease of the primer concentration from 233 nM to 217 nM led to an increase of the measured s/as-ratio of probe pair 1 by factor 200. The hybridization behavior of other PCR-products was tested as well, including a section of the gen *sul1* with 5 probe pairs and the gen *aac(3)-Ia* with 6 probe pairs. These experiments yielded highly similar results (data not shown): At high primer concentrations, the relative position of a probe determined its s/as-ratio (cf. Figure 3 A), and at relatively low primer concentrations, all probes performed qualitatively uniformly, regardless of their particular position (cf. Figure 3 D). The cause of the signal shift from one probe configuration to the other could not be explained with current literature.

**Figure 5:**
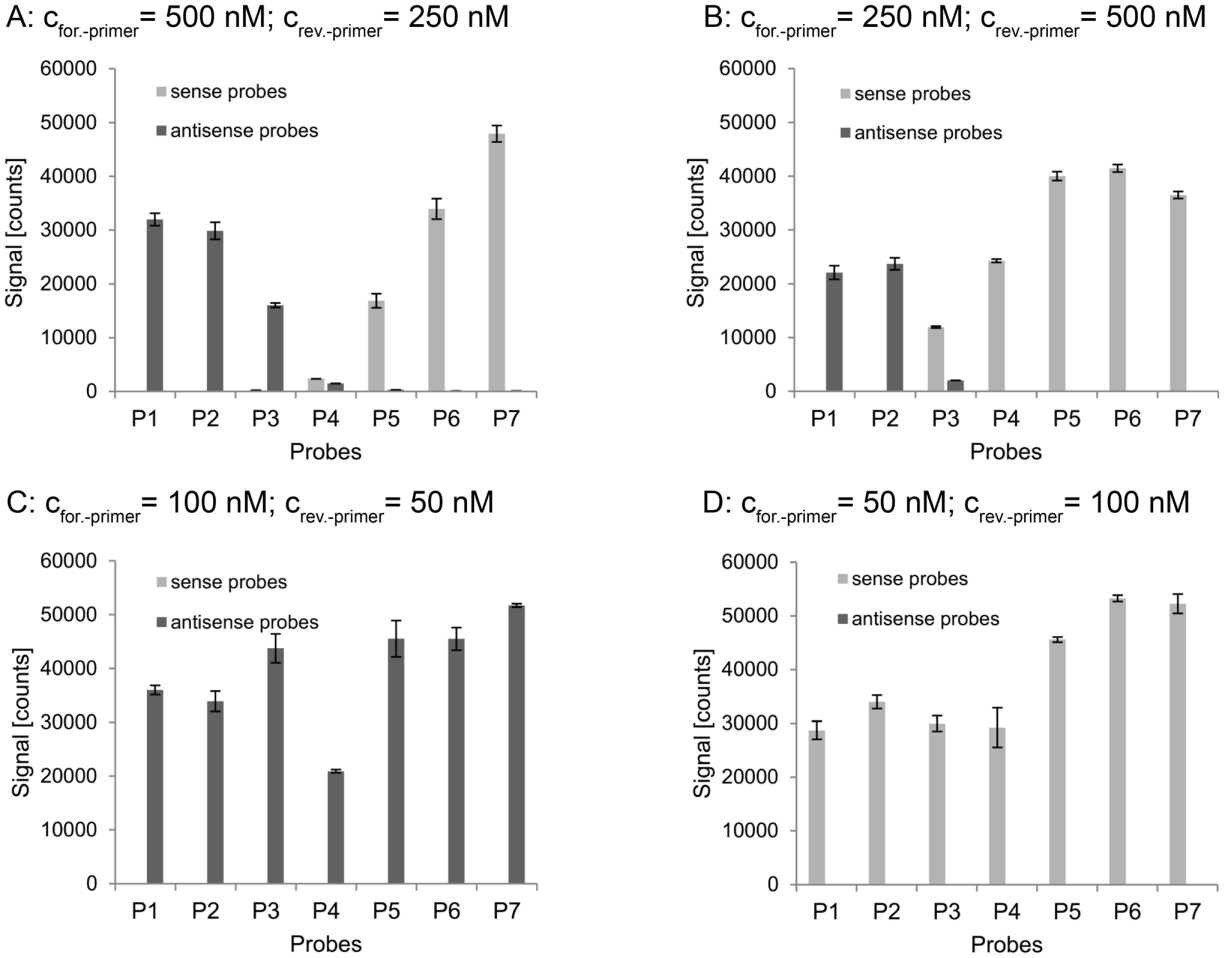
Hybridization of products of four asymmetric PCRs to seven probe pairs. Signal intensities of probe pairs 1–7 after hybridization of the target *dfrA1* which was amplified in four asymmetric PCRs containing different primer concentrations.

A change of the equimolar primer concentration in the PCR was not supposed to change the product characteristics. Therefore, the drastic changes of the hybridization behavior of the PCR products were surprising. However, the primer concentration does influence the generated quantity of each strand. Dependent on the respective primer characteristics the amplification of one of the two strands may be advantaged under primer limiting conditions resulting in a slightly higher yield of one strand. This led to the assumption that single stranded DNA is involved in the hybridization mechanism of PCR-products. Therefore, we investigated the influence of ssDNA on the hybridization reaction. The *dfrA1* gene was amplified in an asymmetric PCR to generate one particular strand in excess. Tested concentrations of forward and reverse primer were 500 nM and 250 nM, 250 nM and 500 nM, 100 nM and 50 nM, and 50 nM and 100 nM, respectively. Resulting signal intensities after hybridization of the generated products are displayed in Figure 5. Whereas the ratio of forward and reverse primer only slightly influenced the signal pattern when high primer concentrations were used, it showed a significant influence on the signal pattern under primer limiting conditions. These data indicated that ssDNA, which is increasingly amplified in an asymmetric PCR, has a strong impact on the hybridization process.

**Figure 6:**
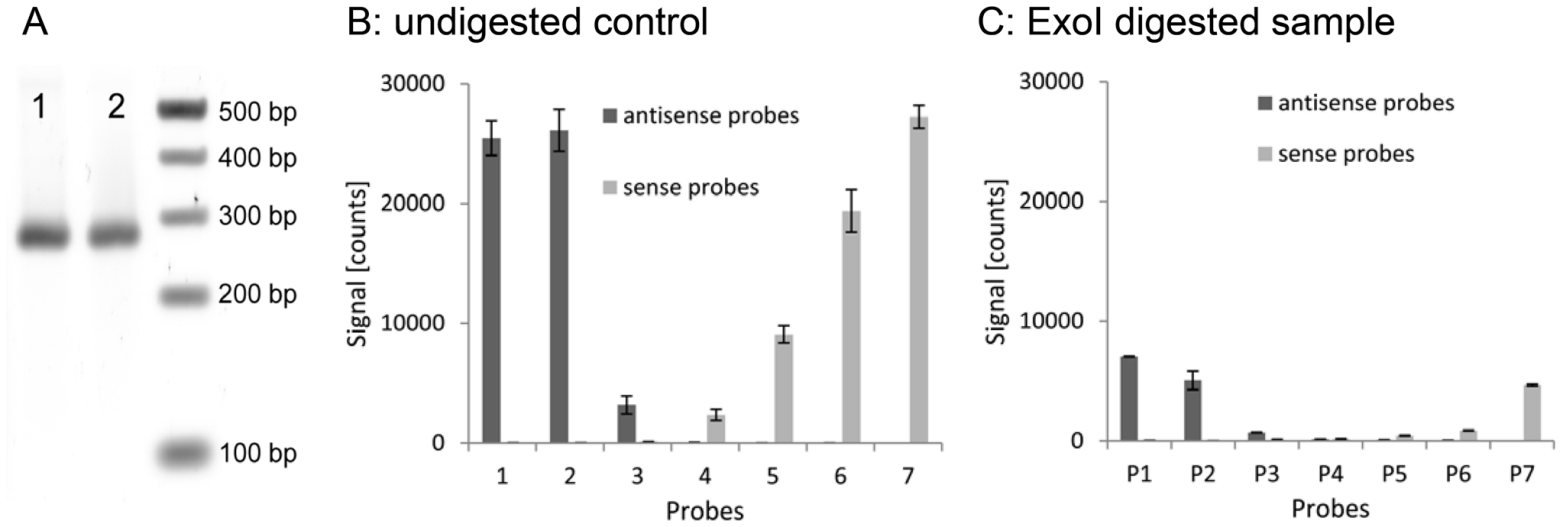
Hybridization behavior of untreated PCR-product in comparison to PCR-product with decreased ssDNA content. (A) gel picture of two samples: untreated *dfrA1* PCR-product (lane 1) and purified and ExoI digested PCR-product (lane 2); (B) Signal intensities of probe pairs 1–7 after hybridization of untreated PCR-product generated with a primer concentration of 250 nM; (C) Signal intensities after hybridization of *ExoI* treated PCR-product. The digested sample was amplified under identical conditions as the control and adjusted to the same concentration after digestion.

In order to further investigate the effect of ssDNA on the hybridization behavior of a PCR-product, we analyzed the PCR-product *dfrA1* with a strongly reduced ssDNA content. The template was amplified with Cy3-coupled primers (250 nM) instead of labeled dCTP and separated by denaturing polyacrylamide gel electrophoresis to get rid of partial amplification products. The band at 268 bases was excised and the DNA extracted. Resulting full-length strands were precipitated with ethanol and digested by the ssDNA-specific exonuclease *ExoI* (250 U/ml) for 72 h. The remaining double-stranded DNA was again precipitated with ethanol before it was hybridized on the array. The result of the PCR-product with depleted ssDNA content was compared to that of an untreated, undigested control sample. Before hybridization, the overall DNA concentration of both samples was measured spectrophotometrically, adjusted to 20 ng/µl, and analyzed by gel electrophoresis using SYBR-gold as staining agent (Figure 6). The reduction of the ssDNA content of the PCR product led to a drastic decrease of the signal intensity of all probes. Only the probes located closest to the ends of the PCR product yielded a small signal.

**Figure 7:**
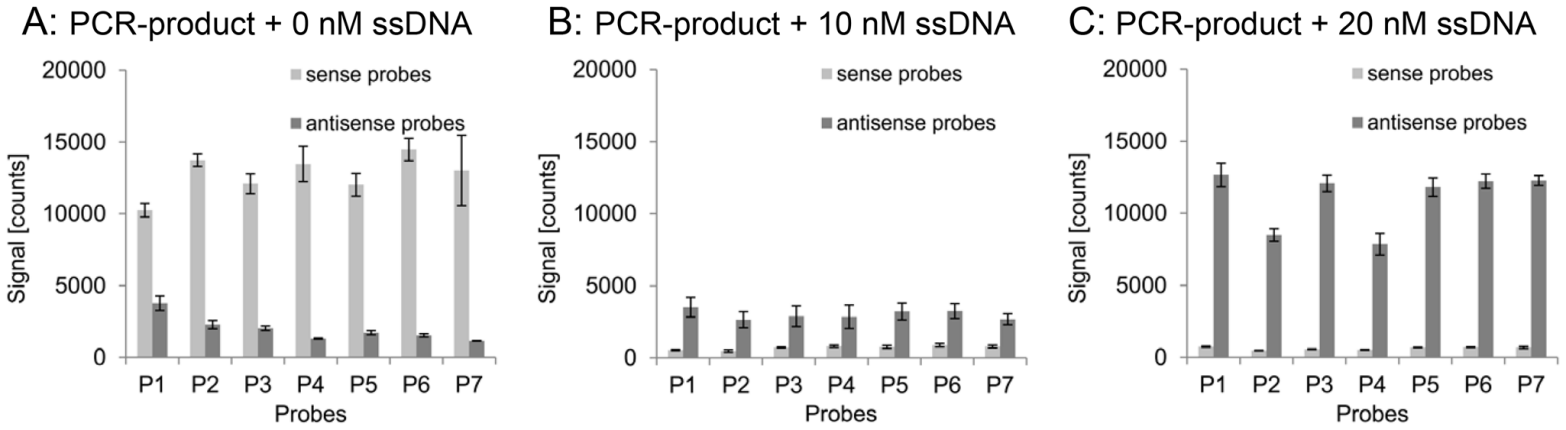
Hybridization behavior of PCR-products supplemented with ssDNA. Signal intensities of probe pairs 1–7 after hybridization of the target *dfrA1* without (A), with 10 nM (B), and 20 nM synthetic not-labeled ssDNA (C). PCR-products were generated with a primer concentration of 125 nM. The addition of synthetic sense strand led to a drastic change of the hybridization pattern by switching the binding preference of the target *dfrA1* to the antisense configuration of each probe.

In an opposing approach, we hybridized the PCR-product *dfrA1* with and without the addition of a defined amount of chemically synthesized ssDNA. This synthetic, unlabeled, 268 bases long strand matched the sequence of the sense strand of the *dfrA1* PCR-product, hence, generating an excess of sense strand in the hybridization mixture. The concentration of Cy3 labeled primers used to amplify the PCR product was 125 nM. One reaction was conducted without and two were supplemented with 10 nM and 20 nM synthetic sense ssDNA, respectively. In contrast to the PCR-products, the synthetic ssDNA was not labeled, and thus, produced no signal on the array by itself. The resulting signal intensities after hybridization are shown in Figure 7. The addition of sense ssDNA led to a massive loss of signal of the sense probes and strongly enhanced the signal of antisense probes. Considering that the additional synthetic ssDNA was not labeled, and thus, had no direct effect on probe signal intensities, the observed signal shift evidenced that the sense ssDNA supports the hybridization of labeled PCR-product to antisense probes. Otherwise, the unlabeled sense ssDNA would have acted as a competitive inhibitor by blocking binding sites of antisense probes.

**Figure 8:**
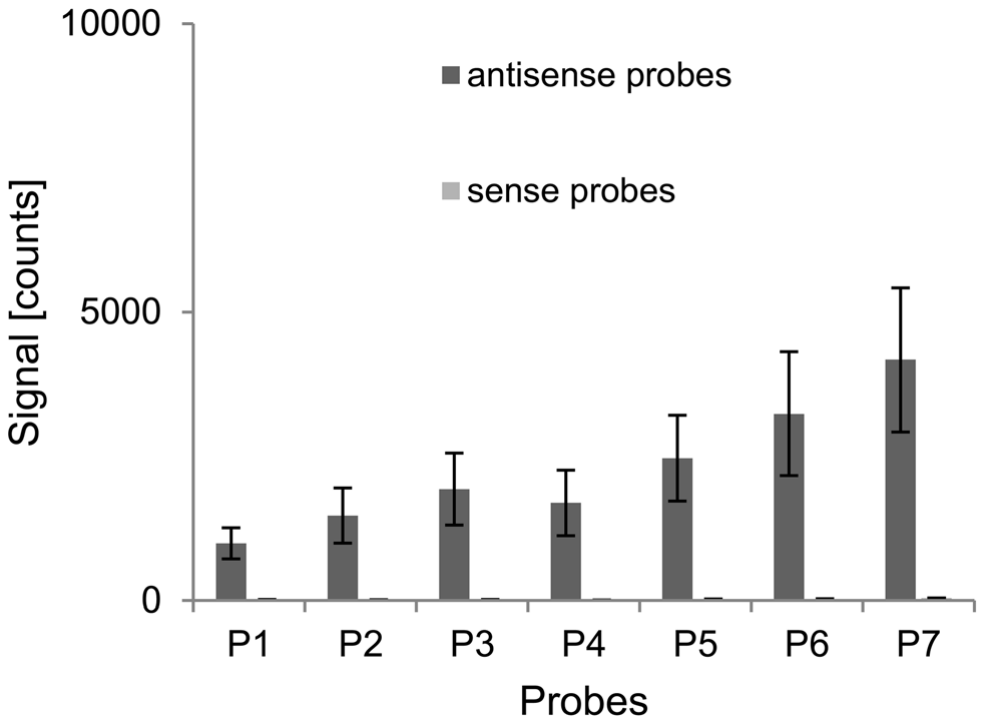
Hybridization behavior of selectively labeled PCR-products supplemented with ssDNA. Signal intensities of the seven *dfrA1*-specific probe pairs after hybridization of the target *dfrA1* consisting of a labeled antisense and a not-labeled sense strand. The PCR-product was generated with a primer concentration of 125 nM and supplemented with 20 nM synthetic sense strand before hybridization.

The total signal of a probe is composed of the signal generated by bound sense and antisense strand. In order to quantify particularly the contribution of antisense strand to this signal, we used a different labeling strategy: only the reverse primer was coupled with Cy3. Consequently, the signal of every probe correlates with the amount of bound antisense strand. The PCR-product *dfrA1* was amplified with a primer concentration of 125 nM and supplemented with 20 nM unlabeled synthetic sense ssDNA before hybridization. The resulting signal intensities are shown in Figure 8. The antisense probes produced a positive signal, although, the labeled antisense strand could not bind directly to them. The binding had to be mediated by the sense strand. This confirmed that not only the target strand of a capture probe, but aggregates of both, sense and antisense strands, were bound to the probes.

## Discussion

We investigated several phenomena regarding the hybridization of PCR-products to microarray probes which could not be explained satisfyingly with existing models. These were the theoretical hybridization mechanism of dsDNA to probes, the configuration selectivity of PCR-products, the switch of this configuration selectivity under certain conditions, and the influence of the probe position score on the s/as-ratio, respectively. Our results indicate that ssDNA plays an important role during hybridization and is the cause of the described phenomena. Single stranded DNA is an often unattended by-product of a PCR and also an ingredient of every hybridization approach which contains PCR-products. Total ssDNA comprises of abortion products, which are generated when the polymerase dissociates from its template during elongation, and full-length products. Single stranded full-length products may exist only of one type of strand at a time because two complementary strands would hybridize with each other. Therefore, only the excess strand is available as ssDNA and can hybridize directly to its corresponding probe.

**Figure 9:**
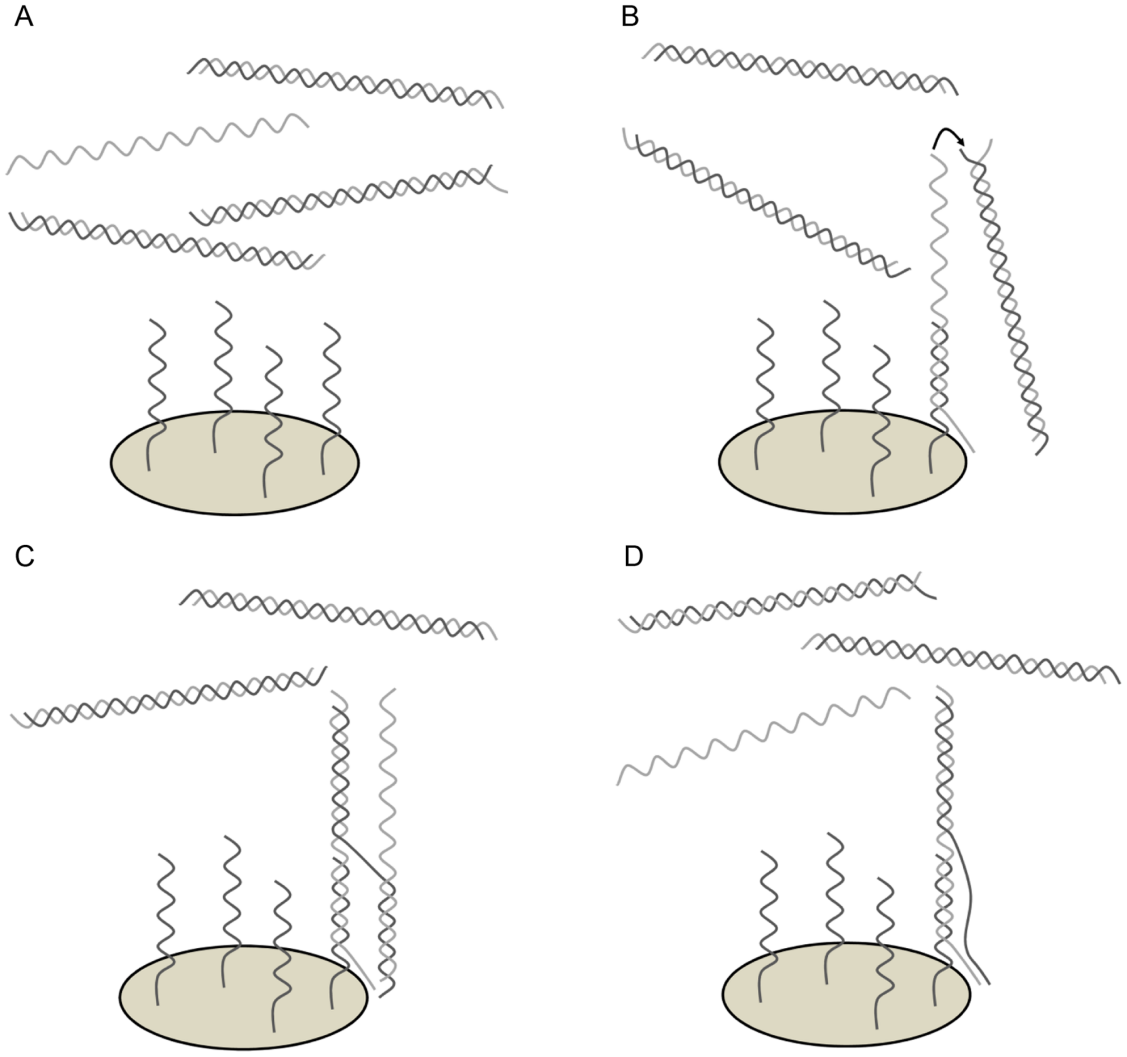
Model for the ssDNA induced hybridization of a PCR-product to microarray probes. (A) PCR-product with an excess of one strand in solution over a microarray probe spot. (B) The ssDNA hybridizes to the probes and its dangling end can interact with the dsDNA. (C) Via a branch migration mechanism one strand of the dsDNA can be transferred to the former ssDNA. (D) When the strand is completely transferred, the laid off complementary strand can bind to a probe again and catalyze the hybridization of another dsDNA.

We developed a new model for the hybridization mechanism which relies on the principle that dsDNA is almost unable to bind to capture probes without ssDNA as catalyst. A possible mechanism of ssDNA induced probe hybridization would be that at first the excess strand hybridizes to its probe. The resulting dangling ends could again act as probes as described earlier [10]. These single stranded dangling ends would have two advantages in binding to double stranded PCR-products compared to covalently bound oligonucleotide probes. On the one hand, they are generally much longer, resulting in a more stable hybridization. On the other hand, their terminal sequence matches the sequence of the end of the PCR-product. Denaturing of a few bases is much more likely to occur at the end of a double stranded PCR-product than in the middle of it. This could enable the dangling tail to attack this end and partially hybridize to its complementary strand. Via a branch migration mechanism the former dangling end could displace the other strand [11]. A complete release of this strand would regenerate the original free ssDNA enabling further reaction cycles. According to the mentioned mechanisms, the displacement of probe bound dsDNA is also possible, so that dissolved and attached dsDNA are in steady state equilibrium after some time. The proposed mechanism shown in Figure 9 is theoretically, and several similar reaction mechanisms are imaginable, such as a further branching of the strand agglomerate shown in Figure 9 C forming branched DNA (bDNA), as observed recently. Aggregates of multiple sense and antisense strands bound to capture probes have been described already [12]. A beneficial effect of increased ssDNA generation on microarray signals, for example by asymmetric PCR, has already been described, too, but the mechanism of action has not been characterized, yet [13]. Another method already in use to increase the ssDNA content in the hybridization mixture is to initially separate the strands of the PCR-product by heat denaturation. However, the amount of ssDNA declines over time due to renaturation of both strands.

Our experiments indicate that ssDNA strongly enhances the hybridization of PCR-products to the probe configuration specific for the ssDNA. The increased generation of one specific strand in an asymmetric PCR and the addition of synthetic ssDNA, respectively, had led to drastic changes of the s/as-ratio of some probes (cf. Figure 5 and Figure 7). According to our model, the addition of synthetic sense ssDNA increased the signal of antisense probes by providing a catalyst for the hybridization and decreased the signal of the sense probe in return by reducing the amount of free antisense strand. The considerable extent of the signal shift supports the assumption that ssDNA is essential for efficient hybridization. In an equimolar PCR, a signal shift of some probes could be observed, too, when the overall primer concentration in the PCR was reduced. The experiments with products of asymmetric PCRs indicated that this effect may be due to minor aberrations in the primer concentrations which become relevant when the primers become a limiting factor in the PCR. It could be observed that a change of the overall primer concentration in an equimolar PCR of 10% may lead to a change of the s/as-ratio of a probe by factor 200 (cf. Figure 4).

**Figure 10:**
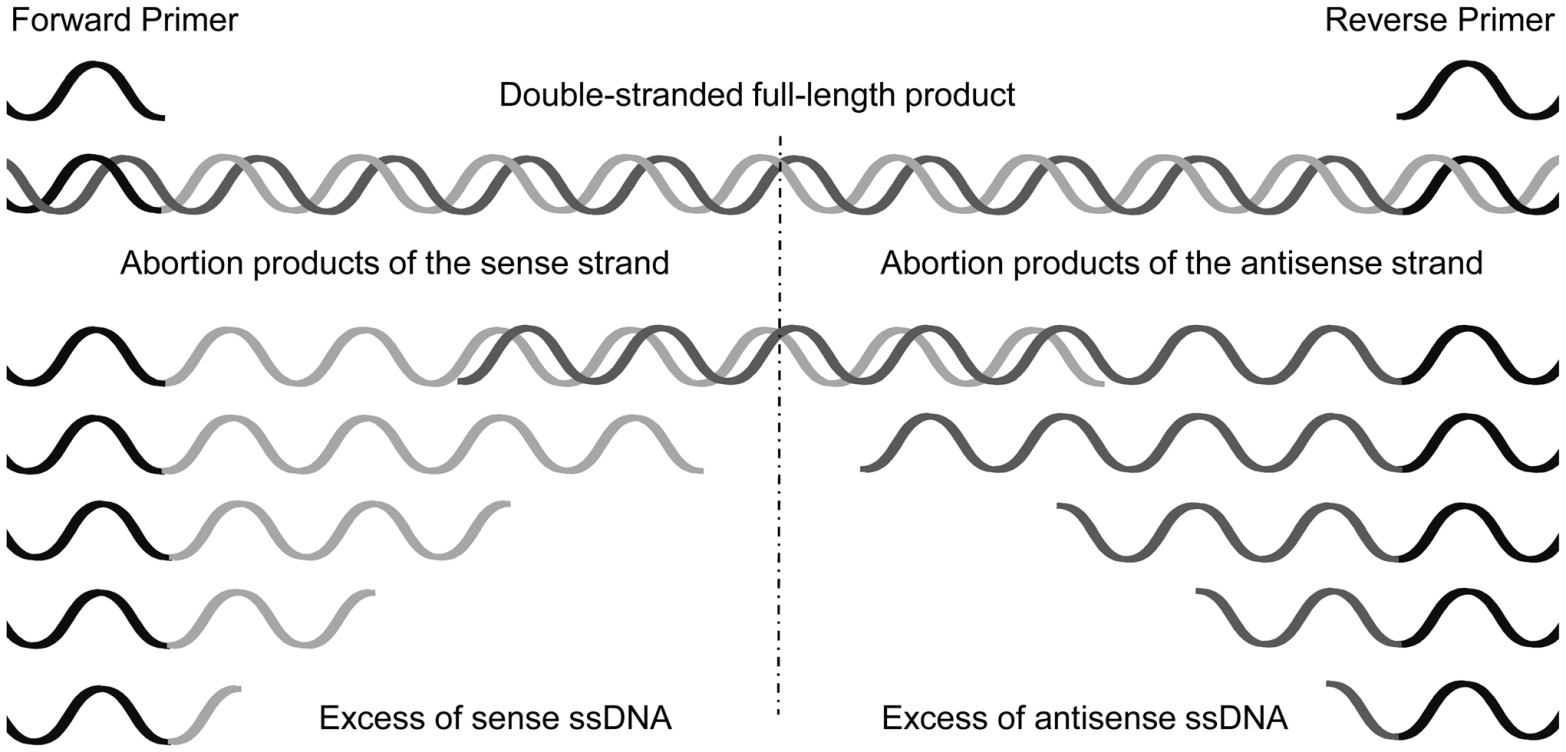
Schematic formation of a double-stranded full-length PCR-product and abortion products of different lengths. The abortion products of the sense strand lead to an excess of sense ssDNA in the vicinity of the forward primer binding site and those of the antisense strand in the vicinity of the reverse primer binding site, respectively. When both strands are formed to the same extend the equilibrium of sense and antisense strands lies in the middle of the product. The formation of one strand in excess leads to a shift of the equilibrium to one side.

It is often reported that the antisense configuration of a probe gives a stronger signal than the sense configuration when the probe is located near the forward primer and the other way round when it is located near the reverse primer (cf. Figure 3 A). This phenomenon is sometimes attributed to secondary structures of the PCR-product or steric hindrance [8]. However, one can also observe that the preference of a PCR-product to hybridize to the sense or antisense probe may change when PCR conditions, especially primer concentration, are modified. Since the structure of a PCR-product does not change when the primer concentration is altered, this phenomenon argues against a major impact of the secondary structure or steric hindrance. However, abortion products, which are generated in every PCR to some extent, may be responsible for the observed phenomena. The fragments of the sense strand support the hybridization to the antisense configuration of probes with a low PPS, and abortion products of the antisense strand support the hybridization to the sense configuration of probes with a high PPS (see Figure 10).

The developed model provides explanations for several unresolved questions regarding the hybridization process of microarrays, such as the theoretical mechanism of the hybridization of dsDNA to probes. It can also clarify why only one probe configuration shows a signal at a time and why this signal may shift to the other probe configuration when experimental terms are modified. Considering the effect of abortion products of a PCR, the model can, furthermore, explain the impact of the relative position of the probe binding site within the target on the probe signal intensity. The proposed hybridization mechanism needs to be confirmed experimentally but several data argue for it. Our results show that ssDNA supports the hybridization of PCR-products to capture probes, which is the basis of the developed model. These findings allow a deeper understanding of the molecular mechanism of probe hybridization. Current mathematical models for the analysis of DNA-microarray data do not consider the influence of ssDNA [14], [15]. Enhancing these models by implying the influence of ssDNA may increase their accuracy. For economic reasons, it is common practice during the development of a microarray to eliminate one configuration of a probe when the other one yields higher signals. This practice becomes problematic when the PCR-protocol is modified. Even minor changes may cause a switch of the probe selectivity of some targets which would result in extremely low signals of the remaining probe configuration. Especially probes showing a s/as-ratio close to 1 react very sensitively to minor aberrations in the experimental setting which affect the ssDNA yield. On the other hand, the high sensitivity of these probes might be utilized in an assay to detect even smallest amounts of ssDNA. The targeted use of ssDNA as hybridization enhancer may improve present diagnostic systems and simplify the development of further methods. In the past unpredictably fluctuating and missing signals have led to considerable frustration of scientists working with microarrays. By using our model, these effects can be explained and strategies can be derived to eliminate their causative origin. Thereby, it should be possible to design more reliable microarray-based analyzing systems.
